# Disclosure and Experiences of HIV-Related Stigma among Adolescents and Young Adults Living with HIV in South Africa

**DOI:** 10.1007/s10461-024-04487-9

**Published:** 2024-09-09

**Authors:** Johanna Nice, Tonya R. Thurman, Brian Luckett, Babalwa Zani

**Affiliations:** 1 Highly Vulnerable Children Research Center, Tulane University School of Public Health and Tropical Medicine, New Orleans, LA USA; 2 Department of International Health and Sustainable Development, Tulane University School of Public Health and Tropical Medicine, New Orleans, LA USA; 3 Tulane International, Cape Town, South Africa

**Keywords:** HIV, Adolescents and young adults, South Africa, Stigma, Disclosure

## Abstract

Social networks expand rapidly in adolescence, increasing HIV status disclosure considerations and concerns for young people living with HIV, especially in settings where HIV-related stigma is prevalent. This study examines HIV disclosure and enacted stigma among adolescents and young adults living with HIV in South Africa. This study uses survey data from a sample of 1186 youth living with HIV, aged 14–24, and enrolled in peer support groups led by community-based organizations in KwaZulu Natal and Gauteng provinces, South Africa. Study participants completed a questionnaire on sociodemographic details, physical health, school attendance, who knew the individual’s HIV status, and experiences of HIV-related mistreatment. Mixed effects logistic regression examined the association between experiences of HIV-related mistreatment and factors that may inadvertently disclose one’s status, such as poor physical health and missed school, and knowledge of an individual’s HIV-positive status by their caregiver, household, friends, educators, and most recent sexual partner. Almost a quarter of the sample reported an experience of HIV-related mistreatment in the past six months. After controlling sociodemographic characteristics, missed school due to illness (AOR = 1.75, 95% CI = 1.27–2.43), and knowledge of HIV status by non-family members (AOR = 2.19, 95% CI = 1.60-3.00) were significantly associated with HIV-related mistreatment. Findings suggest that experiences of enacted stigma are common among youth and linked to poor physical health and knowledge of HIV status outside the family. Effective community-level stigma reduction interventions are urgently needed. In the meantime, adolescents need individualized disclosure counseling and support managing their physical health to prevent further inadvertent disclosure and discrimination.

## Introduction

More than 1.7 million adolescents are living with HIV, including approximately 320,000 in South Africa [1]. Despite improved HIV survival and widespread stigma reduction campaigns [2, 3], stigmatizing attitudes toward people living with HIV in sub-Saharan Africa are pervasive [4]. HIV epidemic control efforts highlight HIV-related stigma and discrimination as significant impediments to achieving the UNAIDS 95-95-95 treatment goals [5]. Among young people living with HIV in South Africa, HIV-related stigma has been linked to reduced retention in HIV care [6] and lower ART adherence [7].

Stigma is a multidimensional construct involving the social devaluation of a person based on an attribute associated with negative public attitudes [8]. Enacted stigma is perpetrated by others and involves the direct experience of devaluation, discrimination, or mistreatment based on one’s HIV status [9]. An experience of enacted stigma is thus typically predicated on external awareness of one’s serostatus and is intimately linked to whether one chooses to tell others or not [10]. Nondisclosure has been hypothesized as a manifestation of anticipated or perceived stigma and linked to poorer ART adherence through a lack of social support [11] and concealment of status [12]. While limiting disclosure may restrict available support networks for managing HIV care, disclosure—voluntary or otherwise—may increase the risk of discrimination and mistreatment [11]. Common disclosure concerns of youth include stigma, discrimination, rejection, unauthorized disclosure of their status and questions regarding how HIV was acquired [13].

Adolescents are also concerned with inadvertent, accidental, or deductive disclosure of their status. Secrecy and concealment of status are commonly cited barriers to ART adherence among sub-Saharan youth [14]. For example, South African youth living with HIV report concerns that peers may notice their frequent absences from school due to clinic visits [7, 15], which in turn is associated with their reduced retention in care [15]. Unintentional HIV disclosure can also result from changes in one’s physical appearance, such as weight loss or exhibiting symptoms or disabilities associated with HIV or AIDS [16, 17]. A study among adolescents living with HIV in South Africa found a direct pathway between HIV-related disability and enacted stigma [18].

Despite stigma’s role in hindering optimal HIV care and treatment outcomes [12, 19, 20], few quantitative studies have explored the factors, including disclosure, associated with specific dimensions of stigma among adolescents and young adults living with HIV in sub-Saharan Africa [21–23]. Research in Kenya found higher reported enacted and internalized stigma among youth who had disclosed their status versus those who had not [22], while a study among adolescents living with HIV in Kenya and Uganda found no independent association between perceived stigma and self-disclosure to peers [23]. A more recent study in Kenya found that being in a sexual relationship was associated with both disclosure concerns and enacted stigma [21].

The purpose of this study is to explore whether HIV status disclosure outside the household and factors that may inadvertently disclose an adolescent’s status, such as poor health and missed school, are associated with recent experiences of enacted stigma in a sample of South African youth living with HIV.

## Methods

### Study Design and Population

A cross-sectional survey was conducted from June 2019 to March 2020, with participants enrolling in a structured support group for adolescents living with HIV (Vhutshilo 3) provided by community-based organizations serving orphans and vulnerable children and youth. Eligibility criteria included youth aged 14 and older, living with HIV, aware of their status, enrolled in the support group intervention, and willing and able to give consent for study participation. Program recruitment occurred separately from study recruitment. All program sites in urban communities of Gauteng and KwaZulu-Natal provinces with experience serving youth living with HIV and conducting structured interventions were eligible for inclusion in the study (*n* = 38 sites). Within each eligible site, all members of support groups that commenced operation between June 2019 and March 2020 were invited to participate in the baseline survey (*n* = 128 groups). Further information about the intervention and associated follow-up study is available elsewhere [24].

### Data Collection Procedures

A one-hour survey was administered in English to each group after one of their initial support group sessions by trained staff from an external South Africa-based research organization. The research staff read each question aloud to the group, and participants self-reported their answers in writing using a printed questionnaire. All participants were expected to understand English as the language of instruction for the intervention under study and due to its common use in South African public schools. However, to minimize language barriers, research staff were linguistically representative of the communities they worked in. They offered clarification upon request using a reference copy of the questionnaire locally translated into isiZulu and Setswana, common languages in the study areas. The questionnaire included sociodemographic details, school attendance, physical health, which relationship types know the individual’s HIV status, and experiences of HIV-related discrimination. Participant seating was spaced out to ensure the confidentiality of responses. Confidentiality was also maintained by using coded identifiers, and the participant sealed each completed questionnaire in an individual envelope before returning it to the research staff. Participants received refreshments and a small monetary incentive (ZAR 20, approximately US$1.20) as a token of appreciation upon completing the survey.

### Ethical Considerations

The study was approved by the Social Behavioral Institutional Review Board at Tulane University in the U.S. (study reference no: 2018 − 1736) and the Pharma Ethics Independent Research Ethics Committee in South Africa (study reference no: 181021579). Written voluntary informed assent and consent for survey participation were sought from all participants; the Tulane and Pharma Ethics review boards granted a waiver of parental permission for adolescents under eighteen.

### Survey Measures

Sociodemographic characteristics. Age, gender, food insecurity, and housing quality were included as *a priori* descriptive and control variables. Studies have shown that self-disclosure increases with age and varies by gender [23, 25]. Food insecurity and housing quality were used as socioeconomic indicators of acute and chronic poverty, respectively. Socioeconomic status is associated with poor health outcomes, and there are also intersectional considerations in that poverty may exacerbate the experience of stigma and discrimination based on health status [26, 27]. Lower housing quality was defined as reporting less than two basic amenities: indoor plumbing, non-dirt-flooring, and electricity. Food insecurity was assessed with one item from the Household Hunger Scale [28] focused on missing a meal due to a lack of food in the household in the past week.

#### Disclosure

A question on disclosure (*Do the following people know you are HIV-positive?* [e.g., caregiver, everyone in the household, any friend, any teacher/principal] was adapted from a survey designed for adolescents living with HIV in the Eastern Cape of South Africa [29], with response options simplified to a dichotomous ‘yes/no’ response. A second question limited to respondents who reported they had ever had sex asked: *The last time you had sex*,* did your partner know your HIV status? (Y/N).* Responses to these questions were combined into one binary measure of ‘any’ disclosure to non-family, in which knowledge of the respondent’s HIV status by a friend, teacher/principal, or last sexual partner was coded as “1” and no knowledge of the respondent’s HIV status by a friend, teacher/principal or last sexual partner was coded as “0”.

#### Self-Rated Health and Missed School

Physical health was assessed with a single item on self-rated health over the past month: *How has your overall health been in the last month (30 days)*? (poor, ok, good, very good). Missed school due to illness was assessed with a single item: *In the last month*,* have you missed any days of school because you were too sick to attend?* (yes, no, I do not attend school).

#### Stigma

Two items from the enacted subscale of the Adolescents Living with HIV Stigma Scale (ALHIV-SS) [30] were adapted for this survey. The language was simplified, and a six-month time frame was specified: *In the last six months*,* has anyone treated you badly because of your HIV? (Y/N)* and *In the last six months*,* have you lost friends because of your HIV? (Y/N*). Responses to these questions were combined into one binary measure of ‘any’ enacted stigma, in which a “yes” to either of the questions was coded as “1” and a “no” to both questions was coded as “0”.

### Statistical Analysis

Descriptive statistics included frequencies, means, and standard deviations (SD) to characterize the sample.

Mixed effects logistic regression generated crude odds ratios (ORs) by separately regressing each risk factor on an experience of HIV-related mistreatment. This was done to examine the independent association between each of the different relationship types and an experience of HIV-related mistreatment without having to account for co-linearity.

Mixed effects multiple logistic regression was used to identify the odds of HIV-related mistreatment given the increased risk for inadvertent disclosure (i.e., missed school due to illness, poor health) and disclosure to non-family (any friend, teacher/principal, or sexual partner’s awareness of respondent’s HIV status) while controlling for potential confounding variables (age, sex, food insecurity, housing quality). The adolescents sampled were nested in peer support groups. Thus, a two-level random intercept model was fitted using the “melogit” command in STATA v17 (Stata Corporation, College Station, Texas, USA) with support group specified as the random effect.

## Results

### Survey Response Rates

A total of 126 groups participated within the thirty-eight study sites with 1273 out of 1575 eligible participants completing surveys. The final analysis population was further limited to those with no item non-response on age [0], sex [0], housing [0], food insecurity [13], health [12], school attendance [12], disclosure [44] and HIV-related mistreatment [35] variables, for a total of 1,186 records.

### Descriptive Statistics

The average age of the analytic population was 16.8 years, and 63% were female (Table 1). 26% reported food insecurity and 37% reported lower housing quality. Few rated their health as poor (8%), while 30% reported they missed school due to illness in the past month. Another 9% reported they were not currently in school. Just over half of the sample reported that non-family (teacher, friend, or recent sexual partner) were aware of their HIV status. When each relationship was examined individually, 89% (89%) of respondents stated that a parent or caregiver was aware of the respondent’s HIV status. Additionally, two-thirds (66%) of respondents reported that everyone in their household knew their status. Fewer respondents reported that a friend (33%) or teacher (28%) knew their status. Among sexually active respondents (*n* = 477), 49% reported that their most recent sexual partner was aware of their HIV status. Over one-fifth (23%) of respondents reported any experience of enacted stigma in the last six months: 18% of respondents reported they were treated badly because of HIV, while 13% reported they had lost a friend because of their HIV status.

**Table 1:** Sample characteristics for analytic population (*N*=1186)

Variable	*n*	%	Mean (SD)
**Sociodemographic variables**			
Age in years (Mean, SD)			16.8 (1.9)
Female	752	63.4	
Food insecurity	307	25.9	
Lower housing quality (<2 amenities)	438	36.9	
**Health and disclosure variables**			
Poor health	97	8.18	
Missed school due to illness last month	368	30.0	
Not in school	102	8.6	
Parent/CG knows	1049	89.4	
Everyone in the household knows	771	66.2	
Non-family knows	647	54.6	
Any friend knows	389	33.1	
Any teacher knows	323	27.5	
Most recent sexual partner knows (*N*=477)	233	48.9	
**Enacted stigma variables**			
Treated badly due to HIV status	220	18.6	
Lost a friend due to HIV status	153	12.9	
Any HIV-related mistreatment	276	23.3	

### Mixed Effects Logistic Regression (Crude ORs)

Table 2 shows crude and adjusted odds ratios of reporting an experience of HIV-related mistreatment in the past six months. In terms of sociodemographic characteristics, age, and gender were not significantly associated with HIV-related mistreatment. Experiencing food insecurity was associated with more than double the odds of reporting HIV-related mistreatment (OR = 2.46, 95% CI = 1.80–3.35) while reporting lower housing quality was not significantly associated with HIV-related mistreatment.

**Table 2:** Unadjusted and adjusted baseline odds ratios for factors associated with an experience of HIV-related discrimination in the past six months (*n*=1,186)

	Logistic Regression					Multiple logistic regression				
	Crude OR	(95% CI)		*P*-value		Adjusted OR	(95% CI)		*P*-value	
		LI	UI				LI	UI		
**Fixed Effects**										
***Sociodemographic variables***										
Age in years	1.03	0.95	1.12	0.485		1.00	0.91	1.09	0.924	
Female	1.02	0.75	1.38	0.922		0.99	0.72	1.36	0.949	
Recent food insecurity	2.46	1.80	3.35	0.000	***	1.95	1.41	2.70	0.000	***
Lower housing quality	1.09	0.80	1.47	0.586		1.08	0.79	1.48	0.607	
***Health and school attendance***										
Poor health	1.68	1.03	2.74	0.036	*	1.30	0.78	2.16	0.311	
Did not miss school due to illness last month	REF	REF	REF	REF		REF	REF	REF	REF	
Missed school due to illness	2.20	1.61	3.00	0.000	***	1.75	1.27	2.43	0.001	**
Not in school	1.54	0.91	2.61	0.105		1.29	0.74	2.25	0.363	
***Disclosure***										
Non-family knows	2.49	1.83	3.38	0.000	***	2.19	1.60	3.00	0.000	***
Parent/CG knows	0.94	0.58	1.52	0.790						
Everyone at home knows	1.11	0.73	1.37	0.995						
Any friend knows	2.29	1.70	3.08	0.000	***					
Any teacher knows	1.89	1.38	2.58	0.000	***					
Most recent sexual partner knows	1.97	1.40	2.77	0.000	***					
**Random Effect**										
Peer support group						0.41	0.19	0.87		

OR, Odds Ratio; CI, confidence interval; CG, caregiver; ***p<0.001, **P<0.01, *P<0.05

When knowledge of the respondent’s HIV status was examined by individual relationship types, any friend (OR = 2.29, 95% CI = 1.70–3.08), any teacher or principal (OR = 1.89, 95% CI = 1.38–2.58), and most recent sexual partner (OR = 1.97, 95% CI = 1.40–2.77) were each associated with a recent experience of HIV-related mistreatment. Parent or caregiver knowledge of the respondent’s HIV status and household members’ knowledge was not significantly associated with HIV-related mistreatment.

When relationship types were dichotomized into a single variable, non-family knowing the respondent’s HIV status was associated with more than two-fold higher odds of HIV-related mistreatment (OR = 2.49, 95% CI = 1.83–3.38). Factors that may inadvertently disclose one’s HIV status were also associated with HIV-related mistreatment. Respondents who reported poor health had slightly higher odds of experiencing mistreatment (OR = 1.68, 95% CI = 1.03–2.74) than those who reported ok, good, or very good health. In addition, respondents who missed school due to illness in the past month had increased odds of mistreatment (OR = 2.20, 95% CI = 1.61-3.00) compared to those who did not miss school.

### Mixed Effects Multiple Logistic Regression (Adjusted ORs)

In the adjusted analyses, missed school due to illness (AOR = 1.75, 95% CI = 1.27–2.43) and non-family knows HIV status (AOR = 2.19, 95% CI = 1.60-3.00) remained associated with HIV-related mistreatment. Experiencing recent food insecurity was associated with higher odds of HIV-related mistreatment (AOR = 1.95, 95% CI = 1.41–2.70) while reporting poor health was no longer significant. The likelihood ratio test for the mixed-effects logistic regression model gave a chi-bar-squared statistic of 16.41 (*p* < 0.0001), confirming significant intraclass correlation within peer support groups and supporting the inclusion of the random effect.

## Discussion

This study found that almost a quarter of adolescents and young adults living with HIV and enrolled in structured support groups in urban communities in South Africa experienced recent enacted stigma. Results on the magnitude of exposure are comparable to the handful of other studies that have measured experiences of HIV-related enacted stigma among young people in Sub-Saharan Africa [6, 22] and adults in South Africa [2].

Over 90% of adolescents reported that someone else knew their HIV status. Caregivers were the most frequently reported relationship type to be aware of the respondent’s status, which may reflect both the importance of family in pediatric disease management and the presence of perinatally acquired HIV in the sample. Previous research from South Africa has highlighted that caregivers’ knowledge of adolescent serostatus is important for facilitating care [15, 31]. Beyond the family, more than half the sample reported that a friend, teacher, principal, or their most recent sexual partner was aware of their status. Knowledge of one’s HIV status outside of the family or household was associated with double the odds of enacted stigma, even after controlling for factors that may be associated with inadvertent disclosure. In contrast, a study in Kenya found youth who disclosed their HIV status only to family experienced significantly higher stigma compared to those who did not disclose to anyone, while disclosure to non-family members did not show a significant difference [22].

When examined individually, each non-family relationship type – friend, teacher/principal, and most recent sexual partner – was associated with increased odds of HIV-related mistreatment. These findings support East and South African qualitative research highlighting concerns around status disclosure among peer networks and within school environments [7, 32, 33]. For example, adolescents in South Africa report fearing they would be gossiped about and rejected if they disclosed their status to friends [7]. Kenyan students described teachers’ violating confidentiality and experiences of bullying, ridicule, and isolation by school staff and students alike [33].

Only half of the sexually active sample in this study reported that their most recent sexual partner was aware of their HIV status, and awareness was associated with enacted stigma. Other studies have illustrated that disclosure to romantic or sexual partners is among the most challenging types of disclosure and is affected by the HIV status of the partner and the kind of relationship (e.g., casual vs. main) [13]. Among sexually active people in South Africa, disclosure to sexual partners is low, with rates of 23% in a nationally representative sample of people living with HIV age 15 and older [34]. A study focused on adolescents living with HIV in South Africa similarly found rates of only 35% disclosure [25]. As with broader peer networks, evidence suggests that a fear of rejection inhibits sharing one’s HIV status [7, 25].

These results support calls for careful attention to the full consequences of disclosure among young people [13, 25]. During adolescence, peers are an important source of emotional support [35], yet findings suggest that adolescents living with HIV cannot disclose their status indiscriminately. While recognizing the limited evidence base around adolescent disclosure, the World Health Organization recommends that adolescents be counseled and empowered to decide when, how, and to whom to disclose their HIV status [36]. Individualized disclosure counseling by healthcare or social workers may help young people assess and manage the different degrees of risk and benefit involved with disclosure, especially as their social networks expand and change with age. Indeed, youth living with HIV have emphasized the importance of making their own choices around disclosure and requested support to develop skills for decision-making, initiating discussions, and managing adverse reactions [13, 36]. However, providers may experience challenges adequately addressing disclosure with adolescents during limited engagements [36].

Consistent with earlier research examining HIV-related disability in South African adolescents [18], this study found that factors that may inadvertently disclose one’s HIV status, including missing school due to illness, were associated with increased odds of a recent experience of enacted stigma. Self-rated health was not associated with enacted stigma, perhaps because this indicator may, in part, reflect emotional dimensions of well-being [37]. Adolescents who missed school due to illness may experience more enacted stigma than those reporting poor health because their absences are objectively noticeable; this may also explain why food insecurity was associated with increased odds of experiencing HIV-related mistreatment, while housing quality was not. Food insecurity may lead to visible signs of malnutrition, weight loss, and disease progression [38], reinforcing negative stereotypes about people living with HIV being unwell, unable to manage their condition, or impoverished [27].

The physical manifestation of disease often drives the fear that results in stigma; thus, mistreatment may lessen as health improves. At a community level, widespread ART initiation is expected to reduce enacted stigma via the power of healthy appearances [39]. At an individual level, improving food security, supporting adolescents in adhering to ART, and managing side effects and opportunistic infections may ease anxieties around unintentional disclosure by improving health and reducing school absenteeism. Holding clinic appointments outside of school hours may also help reduce school absences and improve retention in care [40]. However, while it is possible that adolescents were mistreated by individuals who associated missed school with HIV-related illness, it is also possible that the reported mistreatment occurred *within* healthcare settings during their clinic visits. Research from South Africa and elsewhere has highlighted the detrimental effects of healthcare discrimination on the continuum of care outcomes among adolescents and adults living with HIV [6, 41, 42].

These findings reinforce the need for broader community stigma reduction. However, rigorous evidence is lacking for HIV stigma-reduction interventions [43–45], particularly among adolescents [46, 47]. In the presence of high levels of stigmatizing attitudes among youth [48], adolescents may need additional social support in more secure networks, such as clinic-based peer support groups [21, 40, 49, 50] and households bolstered by family-centered programming [51]. Research also highlights the promise of adolescent clinics and adolescent-friendly health services in creating a more supportive care environment [21, 52, 53].

This study reached a large community-based sample of adolescents living with HIV in a high HIV prevalence setting. Adolescents were recruited to participate in the survey from peer support groups provided by community-based organizations. They may differ systematically from youth who choose not to or cannot participate in community-based organizations’ interventions. Due to the brevity of the research tool, this study did not include a more extensive assessment of stigma, such as that captured in the Adolescents Living with HIV Stigma Scale (ALHIV-SS) [30], or assess whether the adolescent had intentionally self-disclosed their HIV status, the extent to which disclosure occurred, or whether additional relationship types (e.g., neighbors, church members, work colleagues, or community leaders) were aware of their status. The study also did not measure the mode of HIV transmission, which should be included in future studies on disclosure. Adolescents with behaviorally acquired HIV may experience significant challenges disclosing their status to parents or caregivers [13]. Temporality and causality cannot be established with the cross-sectional survey design. In particular, the time frame for the survey questions on HIV-related mistreatment was six months, while that for health and school absences was only the past month. Even if the time frames were similar, reverse causality could not be ruled out, as an experience of HIV-related mistreatment may have resulted in poor self-rated health and school absences. For example, people who experience HIV stigma are at higher risk for reduced care-seeking behaviors [54–56] and poorer physical health [57]. Future research should use longitudinal mediation studies to establish the temporal relationship and causal pathway between physical health and enacted stigma.

This study contributes to the limited knowledge of HIV-related enacted stigma and its correlates among adolescents and young adults. It adds to the literature by exploring the extent and relationship between HIV status disclosure and the experience of HIV-related mistreatment among a large sample of adolescents and young adults living with HIV in a high HIV prevalence setting. Findings illustrate opportunities to mitigate enacted stigma by supporting adolescents with managing their physical health and disclosure decision-making.
